# The Spontaneous Remission of Recurrent Lymph Node Metastatic Prostate Cancer With Lowering Serum Prostate-Specific Antigen Level

**DOI:** 10.7759/cureus.25333

**Published:** 2022-05-25

**Authors:** Atsuto Katano, Masanari Minamitani, Keiichi Nakagawa, Hideomi Yamashita

**Affiliations:** 1 Radiology, The University of Tokyo Hospital, Tokyo, JPN; 2 Radiation Oncology, Graduate School of Medicine, The University of Tokyo, Tokyo, JPN

**Keywords:** hormone therapy, lymph node metastasis, prostate cancer (pca), radiotherapy (rt), spontaneous remission

## Abstract

The incidence rate of spontaneous remission of malignant cancer is very low. Reports on spontaneous remission in advanced prostate cancer are extremely limited. Our patient was treated with androgen deprivation therapy, local radiotherapy, and surgical castration at the initial diagnosis. Approximately nine years after treatment, he experienced a rise in serum prostate-specific antigen level and relapse of obturator lymph node adenopathy. Initially, androgen deprivation therapy was reinitiated, which resulted in castration-resistant prostate cancer. Although androgen deprivation therapy was discontinued, spontaneous remission of recurrent lymph node and spontaneous reduction in serum prostate-specific antigen level was seen. There was no sign of radiological recurrence for over eight years without prostate cancer treatment.

## Introduction

Prostate cancer was the second most common cancer in men worldwide in 2020 with approximately 1.41 million new cases and 375,000 deaths per year [1]. The age-standardized incidence rate of prostate cancer was 37.5 per 100,000 in developed countries, which ranked second following lung cancer. The risk factors for prostate cancer are genetics, obesity, physical activity, smoking, and dietary habits [2]. A genome-wide association study analyzed the genetic component related to oncogenesis in prostate cancer and revealed that germline variation at 8q24 is a strong risk factor for familial prostate cancer [3]. Germline pathogenic breast cancer gene 2 mutations also increase the risk of prostate cancer [4].

Treatment strategies for prostate cancer include a wide variety of modalities, such as surgery, hormonal therapy, radiotherapy, and chemotherapy [5]. Hormone therapy for metastatic prostate cancer is the most effective treatment; however, most cases eventually stop responding to hormone therapy, resulting in castration resistance. Loffeler et al. reported a median overall survival of 12.3 months for metastatic castration-resistant prostate cancer without life-prolonging treatment [6]. This article reports a rare case of spontaneous remission of recurrent lymph node in castration-resistant prostate cancer. There was no sign of radiological recurrence with a low serum prostate-specific antigen (PSA) level for over eight years with no prostate cancer treatment.

## Case presentation

**Figure 1:**
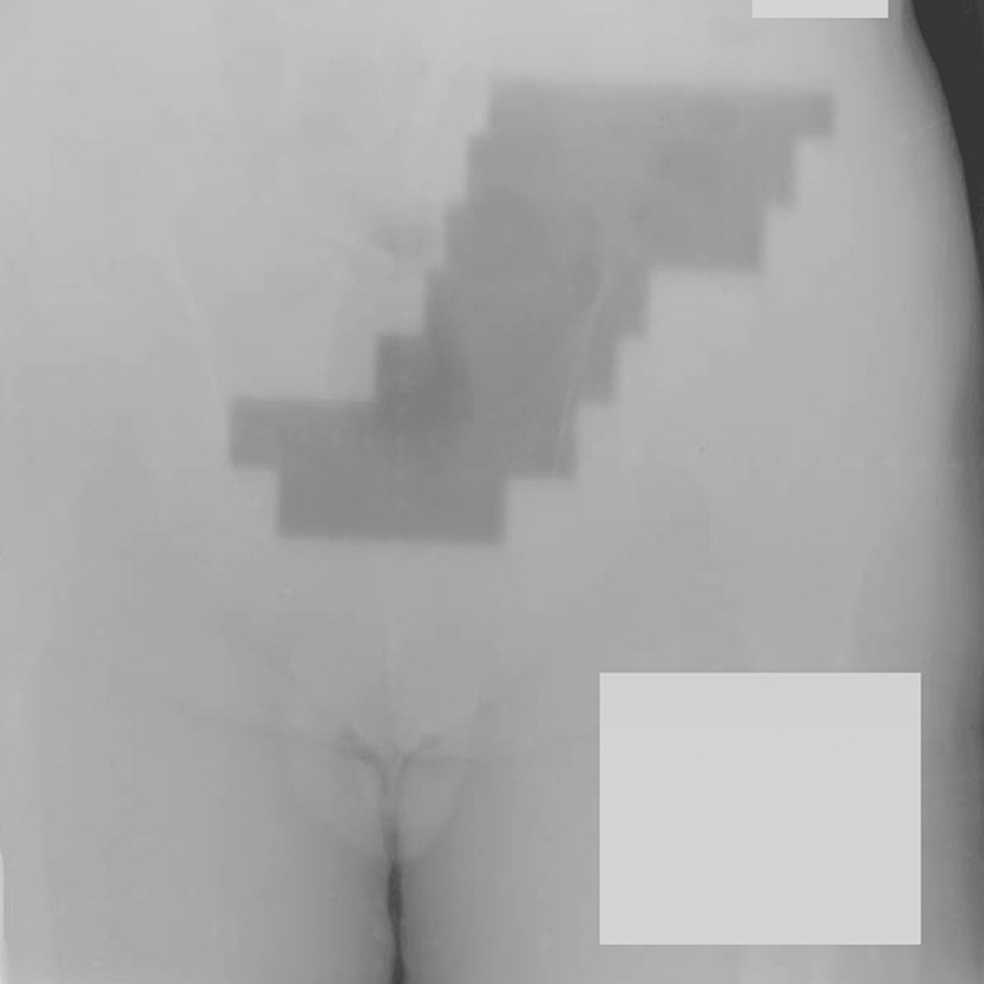
Pelvic treatment fields with two-dimensional conformal radiotherapy (static anterior-posterior/posterior-anterior). The dark area in the center of the image indicates the target of radiotherapy. This treatment field included the left iliac lymph node and bilateral obturator lymph node. The selected energy of photons was 6 megavolt.

A 55-year-old man was referred to our department in September 1997and the Eastern Cooperative Oncology Group Performance Status Scale (ECOG-PS) was 0. The patient was diagnosed with prostate cancer with regional lymph node metastases at a hospital in April 1997. His initial PSA level was >100 ng/mL. Administration of chlormadinone acetate and leuprorelin acetate was initiated in the previous hospital. Chlormadinone acetate is a progestin drug and leuprorelin acetate is an agonist of luteinizing hormone-releasing hormone. Pelvic computed tomography (CT) revealed lymphadenopathy of the left iliac lymph node. The biopsied specimens from the previous hospital were reevaluated at our institution, which revealed a Gleason score of 4 + 5 moderately differentiated adenocarcinoma in five of six cores. He received radiotherapy for the lesion, which consisted of a total dose of 60 Gy in 30 fractions from parallel opposed beams, in October 1997 (Figure 1).

**Figure 2:**
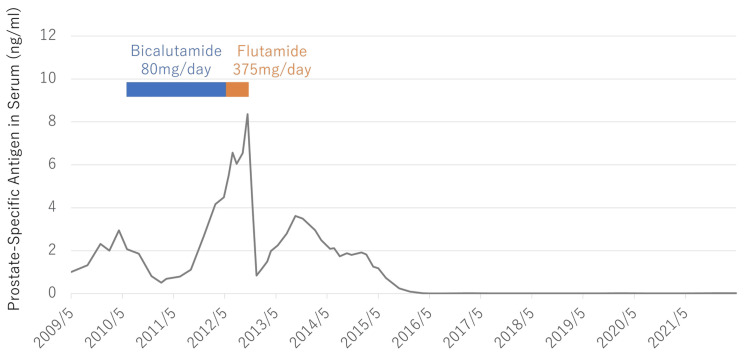
Serum prostate-specific antigen level response and the duration of hormone therapy after relapse.

**Figure 3:**
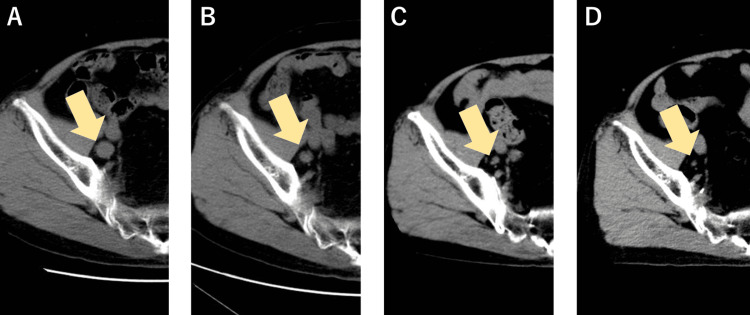
Time course of axial CT scan of the pelvis: (A) November 2012 (PSA:8.36 ng/ml), (B) December 2013 (PSA: 3.5), (C) February 2015 (PSA: 1.8), and (D) August 2018 (PSA: 0.01). PSA: prostate-specific antigen

Surgical castration to remove the testicles was performed after radiotherapy. Thereafter, the serum PSA level decreased to approximately 0.1 ng/ml, which was maintained for nine years without chemical hormone therapy. From 2007, his serum PSA level gradually rose and reached 2.95 ng/ml in April 2010. He was started on androgen deprivation therapy with bicalutamide (80 mg/day), an androgen receptor antagonist. His serum PSA level decreased to a nadir of 0.51 ng/mL in February 2011 and then rose to 4.49 ng/ml. Androgen deprivation therapy was switched to flutamide (375 mg/day:125 mg thrice daily), an androgen receptor antagonist, in June 2012; however, the PSA level increased to 8.36 ng/ml. Swelling of the right pelvic lymph nodes was detected using CT imaging in November 2012. Therefore, we considered him to have castration-resistant prostate cancer, according to the definition of the Canadian Urologic Oncology Group and the Canadian Urological Association [7]. He was discontinued on flutamide, preparing for cabazitaxel administration; however, his PSA levels decreased (Figure 2), and CT scan showed a trend toward shrinkage of the pelvic lymph nodes (Figure 3).

**Figure 4:**
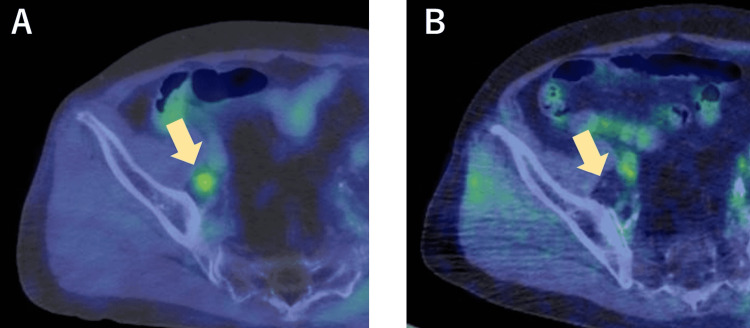
Comparison of 18F-fluorodeoxyglucose accumulation at recurrent lymph node by PET-CT images: (A) April 2012 (Maximum standardized uptake value: 3.6) and (B) April 2020. PET-CT: Positron emission tomography-computed tomography

Subsequently, PSA remained at a low level for over eight years without any prostate cancer treatment, including androgen deprivation therapy, and there was no sign of radiological recurrence. The accumulation of ^18^F-fluorodeoxyglucose at the right pelvic lymph node disappeared, which was confirmed by positron emission tomography-computed tomography (PET-CT) (Figure 4). He maintained his ECOG-PS of 0.

## Discussion

In the current case, hormone therapy was selected as the initial treatment because lymph node metastasis was present at the time of diagnosis. Swanson et al. reported the five-year and 10-year survival rates of lymph node-positive prostate cancer were 78% and 56%, respectively [8]. Our patient is still alive 25 years after the initial diagnosis and is not currently receiving any treatment.

The incidence rate of spontaneous remission of cancer is extremely low and is reported to occur in approximately one in over 100,000 cancer patients [9]. Chodorowski et al. reported that spontaneous remission is often reported in neuroblastoma, renal cell carcinoma, malignant melanoma, and lymphoma [10]. To the best of our knowledge, spontaneous remission of prostate cancer is rare. Yan et al. reported a complete remission case of metastatic prostate cancer after discontinuation of oral flutamide administration [11]. Lee et al. reported that PSA level decreased spontaneously in recurrent lymph node metastasis after radical prostatectomy [12]. The patient presented with a spontaneous drop in serum PSA level and shrinkage of the left internal iliac node adenopathy approximately two years after prostatectomy without hormone therapy. 

The mechanisms of spontaneous remission are associated with composite factors including the host immune system and tumor microenvironment [13]. The host immune system is modulated by the response to microbial infection, surgical intervention, physical trauma, and psychological factors [14,15]. Shibata et al. suggested an antineuronal antibody, anti-Hu, in cases of spontaneous remission of small cell lung cancer [16]. However, further research is required to investigate the detailed mechanism of spontaneous remission.

## Conclusions

We presented a rare case of spontaneous remission of recurrent metastatic lymphadenopathy in prostate cancer. Reports of spontaneous remission in advanced prostate cancer are extremely limited. The insights gained from this case report may have interesting implications for developing cancer treatment. A spontaneous cancer remission is a rare event; however, its oncological mechanism is worth studying for future cancer treatment.
